# Anticholinergic Toxicity Secondary to Overuse of Topricin Cream, a Homeopathic Medication

**DOI:** 10.7759/cureus.2273

**Published:** 2018-03-05

**Authors:** Michael Krzyzak, Angela Regina, Raphael C Jesin, Liliane Deeb, Eric Steinberg, Nima Majlesi

**Affiliations:** 1 Department of Medicine, Staten Island University Hospital, Northwell Health; 2 Emergency Medicine, St Barnabas Health System; 3 Internal Medicine, Holyoke Medical Center; 4 Department of Gastroenterology, Staten Island University Hospital, New York; 5 Emergency Medicine, Mount Sinai Beth Israel Medical Center; 6 Emergency Medicine, Staten Island University Hospital

**Keywords:** anticholinergic toxicity, homeopathic, alternative medicine

## Abstract

Adverse reactions from over-the-counter medications present a challenge to physicians. Homeopathic medicine is an alternative practice, originating in Germany and gaining popularity in the United States. It utilizes dilute preparations of substances in order to treat and cure disease. Patients may potentially suffer serious effects from the use of these products as the contents and concentrations are often unclear. Here, we describe a case of suspected atropine toxicity due to the overuse of a topical homeopathic cream, Topricin, which contains belladonna, a plant containing atropine.

## Introduction

Atropine is a reversible antagonist of muscarinic anticholinergic receptors. It causes higher firing of the sinoatrial node, decreases bronchial secretions, and decreases firing of the vagus nerve. It has several medicinal uses, including treatment of severe bradycardia, anesthesia adjunct to dry oral secretions, and organophosphate and carbamate poisoning [1]. Different forms of plants and herbs that have atropine-like properties are also used in homeopathic remedies like Topricin, a topical preparation to treat pain [2].

Homeopathy relies on the principle of “similars”, which attempts to use one compound to treat the entire host of symptoms. In 1796, Samuel Hahnemann proposed ‘like may be cured with like’. This statement means that a disease that is cured by a remedy whose drug picture is most similar to the disease itself. This pharmacotherapy relies on exact observation and experience rather than to evidence-based medicine [3]. The manufacture of homeopathic medicine involves dilution of an extracted substance in decimal and centesimal stages. The dilution level is based on the experience of homeopathy and on a patient’s symptoms [3]. Newer agents involve combinations of different diluted substances in small quantities, as in the case of Topricin [2].

Homeopathic medicine use should be a concern to a clinician when evaluating for possible drug side effects. Approximately one million children and five million adults use some sort of homeopathic substances in the United States [4]. The United States Food and Drug Administration has regulated substances sold as homeopathic remedies since 1988. Substances for self-limiting conditions can be sold over the counter, but substances for more serious conditions need to be prescribed by a homeopathic clinician [5].

Herein, we report a case of anticholinergic toxicity secondary to a topical homeopathic pain relief remedy.

## Case presentation

A 40-year-old man presented with family to the emergency department complaining of headache and blurry vision of one day's duration. His wife also noticed a change in mentation for two hours prior to presentation when he repeatedly asked the same questions and did not recognize family members. His past medical history included hypertension and dyslipidemia. His daily medications were pravastatin, metoprolol, enalapril, and non-steroidal anti-inflammatories for musculoskeletal pains. Social history and family history were non-contributory. Two days prior to presentation, he had developed severe pain in his left foot which he treated with over-the-counter Topricin (topical pain relief) cream. He applied the cream liberally without respecting the recommended dosage. The exact quantity and frequency were unknown.

On physical exam, his vital signs were significant for sinus tachycardia at 135 beats per minute but otherwise was normal without fever. He was lethargic, yet arousable; his orientation could not be assessed. His pupils were small and no gross focal motor deficits could be elicited. A stroke code was initially called in the emergency room due to his altered mental status, and a subsequent computed tomography scan was negative for any intracranial pathology. Thrombolytics were not given. Laboratory studies showed hypercholesterolemia with a low-density lipoprotein of 114 mg/dL and a creatine kinase (CK) initially of 98 IU/L, which subsequently rose up to 1,837 IU/L on repeat lab testing. A troponin level and creatine kinase muscle-brain (CK-MB) were within normal limits. Urine drug screen was negative. Blood urea nitrogen and creatinine were 29 mg/dL and 1.59 mg/dL, respectively.

In the emergency department, the patient was disoriented and incontinent to urine. A Foley was placed and drained 950 mL of clear urine in one hour, with his incontinence likely representing overflow. Lorazepam was administered for acute delirium. A toxicology consult was obtained. At that point, the possibility of anticholinergic toxicity secondary to the overuse of the Topricin cream was suspected, given the favorable complex of presenting signs and symptoms. A review of the ingredients revealed belladonna. Physostigmine was then recommended as a diagnostic tool. However, due to the recent use of a benzodiazepine and the lack of imminent need, a decision was taken to withhold. The patient was admitted for observation and intravenous (IV) hydration. On the second day, his mental status had returned to baseline, his kidney functions improved, and his CK level approached normal limits. He was discharged home after extensive education regarding the use of over-the-counter and homeopathic remedies.

## Discussion

Topricin is a topical homeopathic cream used for rapid relief of pain and stiffness. It is comprised of the following active ingredients: Aesculus hippocastanum, Arnica montana, belladonna, Crotalus horridus, echinacea, Graphites, Heloderma, Lachesis mutus, Naja tripudians, Rhus toxicodendron, and Ruta graveolens. The belladonna concentration is “6X in 170 g” of Topricin [2]. Belladonna is a component isolated from the herbaceous plant Atropa belladonna. The name "belladonna" is derived from Italian and means "pretty woman" because the herb, which has anticholinergic properties, was used in eye-drops by women to dilate the pupils of the eyes to make them appear seductive [6]. It is commonly used as an herbal remedy to combat pain, reduce secretions, and alleviate muscle spasms. In large quantities, belladonna can induce anticholinergic toxicity. Patients with anticholinergic toxicity may present with flushing, dry skin and mucous membranes, mydriasis with loss of accommodation, altered mental status, fever, sinus tachycardia, decreased bowel sounds, functional ileus, urinary retention, hypertension, tremulousness, and myoclonic jerking leading to rhabdomyolysis. As little as 2 mg of atropine ingestion has been reported to produce those symptoms [7].

Identifying a patient with anticholinergic toxicity can be difficult since its presentation is often similar to delirium caused by other conditions (e.g., infections, substance withdrawal, metabolic disturbance). The correct diagnosis of anticholinergic toxicity depends on the treating physician's awareness of the condition and recognition of its symptoms. Diagnosis of atropine toxicity is mainly clinical. 

Some commonly prescribed medications have detectable anticholinergic effects. Beta blockers, such as the metoprolol which our patient was prescribed, are known to have low anticholinergic activity [8]. On the other hand, the temporal relationship between the institution of Topricin and symptoms, imply it as the sole agent. In addition, metoprolol has minimal anticholinergic properties; thus, it is unlikely to play a role in the patient’s presentation. Further studies are needed to explore the relationship between prescribed medications with anticholinergic activity and atropine-containing medications.

Treatment of anticholinergic toxidrome is based on the severity of the symptoms. Mild cases can be treated supportively, and benzodiazepines can be used for agitation, as they were used in this case. However, the definitive antidote for anticholinergic toxicity is physostigmine, which is used in moderate to severe cases to reverse the central effects of coma, seizures, severe dyskinesias, hallucinations, and severe agitation [9]. Gastrointestinal decontamination may be necessary after anticholinergic poisoning by ingestion.

The patient improved significantly with supportive treatment. Although our case lacked a blood test and physostigmine trial for a definitive diagnosis, the presenting symptoms of delirium, antimuscarinic effects, and the elevation of creatine kinase secondary to muscle breakdown are hallmarks of atropine toxicity. In addition, the possibility of surreptitious ingestion also was considered, although the family stated they witnessed the patient apply this topically. 

Although often advertised as “natural and safe”, adverse reactions to homeopathic treatments are rare but do exist. Often, they occur due to the lack of proper diagnosis and therapy provided by conventional medicine. A systematic review done in 2012 in the United Kingdom showed the most common adverse reaction of homeopathy was allergic reactions [10]. Adverse reactions to homeopathic therapies are likely to be under-reported and a true incidence is unknown.

## Conclusions

Our case illustrates the importance of emphasizing the side effects of natural homeopathic remedies and over-the-counter preparations. Obtaining a detailed history, especially use of non-prescribed medications, is vital when an overdose of a substance is suspected. Theoretically, the amount of substances in homeopathic medications are intended to contain minimal concentrations. Physicians and pharmacists alike should be aware of alternative medications, including homeopathy, in patients presenting with new and unusual symptoms.

## References

[1] Katzung BG, Trevor AJ (2015). Basic & Clinical Pharmacology, 13th Edition. http://books.google.com/books?id=GTgfBQAAQBAJ&q=Basic+%26+Clinical+Pharmacology,+Thirteenth+Edition&dq=Basic+%26+Clinical+Pharmacology,+Thirteenth+Edition&hl=en&sa=X&ved=0ahUKEwjrl7yzldHZAhVDxGMKHTQqBskQ6AEIJzAA.

[2] (2016). TOPRICIN - aesculus hippocastanum, arnica montana, belladonna, calendula officinalis, crotalus horridus, echinacea, graphites, heloderma horridum, lachesis mutus, naja tripudians, phosphorus, rhus toxicodendron, ruta graveolens, sulfur cream. http://dailymed.nlm.nih.gov/dailymed/drugInfo.cfm?setid=321e83de-71e8-20b4-e054-00144ff8d46c.

[3] Righetti M, Ammon K, Mattmann P, Thurneysen A (2011). Introduction to the speciality of homeopathy – principles and definition. Homeopathy in Healthcare – Effectiveness, Appropriateness, Safety, Costs.

[4] Righetti M (2015). Aims of homeopathy research, 1994 and 2015. Homeopathy.

[5] Podolsky SH, Kesselheim AS (2016). Regulating homeopathic products - A century of dilute interest. N Engl J Med.

[6] Kuhn C, Swartzwelder S, Wilson W (2008). Buzzed. The Straight Facts about the Most Used and Abused Drugs from Alcohol to Ecstasy. Buzzed. The Straight Facts about the Most Used and Abused Drugs from Alcohol to Ecstasy.

[7] Glatstein M, Alabdulrazzaq F, Scolnik D (2016). Belladonna alkaloid intoxication: the 10-year experience of a large tertiary care pediatric hospital. Am J Ther.

[8] Lanctôt KL, O'Regan J, Schwartz Y (2014). Assessing cognitive effects of anticholinergic medications in patients with coronary artery disease. Psychosomatics.

[9] Burns MJ, Linden CH, Graudins A (2000). A comparison of physostigmine and benzodiazepines for the treatment of anticholinergic poisoning. Ann Emerg Med.

[10] Posadzki P, Alotaibi A, Ernst E (2012). Adverse effects of homeopathy: a systematic review of published case reports and case series. Int J Clin Pract.

